# Assessment of the mottling score as a mortality predictor in critically ill patients

**DOI:** 10.1186/cc12155

**Published:** 2013-03-19

**Authors:** E Bastos de Moura, F Ferreira Amorim, C Darwin Silveira, M Oliveira Maia

**Affiliations:** 1 Hospital Santa Luzia, Brasília, Brazil

## Introduction

The use of peripheral perfusion objective parameters to anticipate successful resuscitation in septic shock has been recently investigated [1]. The mottling score, a perfusion parameter used for decades, has been proposed to correlate with septic shock survival [2], and was tested in this study as a clinical tool in predicting mortality.

## Methods

A prospective observational study was conducted, with patients consecutively admitted to a tertiary hospital ICU in Brasília, Brazil. From July 2011 to May 2012, all patients diagnosed with septic shock were enrolled. Demographic data, diagnoses, shock origin and severity scores were recorded. After initial resuscitation, the score was registered in the first 3 days by the same observer, considering the score on the lower limb without an arterial catheter, or the worst between the lower limbs, and the worst in the 3 days. Exclusion criteria were terminal illness with no intervention decision and incomplete data. The scores are pooled in Group 1 (scores 0 and 1), Group 2 (scores 2 and 3) and Group 3 (scores 4 and 5) to compare mortality. Statistical analysis was made using the chi-square test.

## Results

One hundred and seventeen patients were analyzed; 20 were excluded (18 terminal illness, two with incomplete data). Ninety-seven patients were included; the mean age was 72.8 years, mean SAPS II score was 46.8 (SD ± 15.7), mean APACHE II score was 19.2 (SD ± 8.1); mean norepinephrine dose was 1.25 μg/kg/minute; mean length of stay in ICU was 19.2 days (1 to 176); mottling score distribution was: score 0: 41 patients; score 1: 33 patients; score 2: 14 patients; score 3: two patients; score 4: three patients; score 5: four patients. The sepsis origin was as follows: 65, pulmonary (67%); 18, abdominal (18.5%); nine, urinary (9.5%); two, osseous (2%); one, mediastinal; one, skin and soft tissue; and one, central nervous system (1% each). Comparing the mortality in Groups 1, 2 and 3, we found a significant difference (*P *= 0.042), even greater when considering 28-day mortality (*P *= 0.004). The Kaplan-Meier survival method showed *P *= 0.000.

## Conclusion

The mottling score was an objective reproducible system to bedside use and a good predictor of septic shock mortality.
